# Relationship between Quality of Life and Sociodemographic, Physical and Mental Health Variables in People over 65 in the Community of Madrid

**DOI:** 10.3390/ijerph17228528

**Published:** 2020-11-17

**Authors:** Berta Ausín, Alba Zamorano, Manuel Muñoz

**Affiliations:** Personality, Evaluation and Clinical Psychology Department, School of Psychology, Complutense University of Madrid, Campus de Somosaguas, Ctra. de Húmera, s/n, 28223 Pozuelo de Alarcón, Madrid, Spain; azlido@ucm.es (A.Z.); mmunozlo@ucm.es (M.M.)

**Keywords:** gender, mental health, older adults, quality of life

## Abstract

Except in the case of depression, there are few studies that analyze mental health variables related to quality of life (QoL) in people over 65 years of age. The objective of this study is to analyze the relationship between QoL and the following variables: sociodemographic and physical and mental health of people over 65 years of age. The sample was randomly selected and consists of men and women between 65 and 84 years of age (*N* = 555) from the Community of Madrid. Mental disorders were evaluated with the CIDI65+ interview and QoL with the WHOQoL-BREF scale. Means, ANOVA and multiple linear regression analyses were performed. Women have worse QoL than men and QoL worsens with age. The regression model for the dependent variable “WHOQoL BREF Scale” explains 41.43% of the variance (*R*^2^ = 0.413). The variables that have the greatest impact on QoL are as follows: a greater number of physical and psychological symptoms, experiencing financial difficulties and the presence of a psychological disorder, while continuing to work has a positive effect on QoL. Physical and mental disorders have a similar impact on QoL. The presence of a greater number of psychological symptoms (without necessarily fulfilling the criteria of a mental disorder) is a predictive variable of worse QoL. Mental health has a burden on the QoL of people over 65 years of age that is as powerful as physical health.

## 1. Introduction

The WHO defines quality of life (QoL) as “an individual’s perception of his or her place in the world, the cultural context and value system in which he or she lives, and all of this in relation to his or her expectations, norms and concerns. It is a broad concept that is influenced in a complex way by the physical health of the subject, his psychological state, level of independence, social relations, and relationship with the essential elements of his environment” [1]. QoL usually appears as a multidimensional construct that includes medical as well as psychological and socio-economic factors [2]. The WHO’s Quality of Life group [3] establishes the existence of six areas or domains of quality of life: physical aspects (which would include facets such as pain, tiredness and sleep), psychological aspects (such as self-esteem, concentration and body image), the degree of independence (with facets such as mobility, work capacity or activities of daily life), social relations (social support, personal relations, etc.), the environment (financial resources, transport, health and social care, etc.) and the spiritual sphere.

In the area of older people, the literature tends to indicate that women have a worse QoL than men [4,5]. Some authors argue that these differences may be mediated by sociodemographic and socio-economic factors, such as income level or marital status [6], as well as by some lifestyle factors, such as a greater tendency of women to lead sedentary lives and a greater presence of obesity [7]. Regarding the impact of age on QoL, data from the English longitudinal study of aging [8], carried out in England with 11,234 people over 50 years of age, indicate that QoL reaches its maximum level at 68 years of age, at which time it begins to decrease, being significantly lower at 75 years of age. These results are in line with the findings of other authors [9]. The decrease in QoL at older ages (75–80) seems to be related to greater functional difficulties in this age group [10].

CoV is not only related to age and sex, but also to physical and mental health [8,11]. Except in the case of depression, few studies analyze the variables of Daniels [11]. In this sense, depression is associated with a decrease in the QoL of older people. Sivertsen et al. [12] carried out a systematic review in this area, including 74 studies (52 of them transversal and 22 longitudinal) that point out the association between depression and low QoL. Chang et al. [13] showed that even mild depressive symptoms have a significant impact on QoL in older people. Jia and Lubetkin [14] obtained similar results in their study in the United States, pointing out that mild depression is associated with a loss of QoL-adjusted years of a similar magnitude to suffering from diabetes or heart disease. Van der Weele et al. [15] showed that 90-year-olds with depression had not only a worse QoL but also a higher risk of mortality. In the case of anxiety, people over 65 years of age who suffer from anxiety show reduced QoL [16,17]. On the other hand, studies on the impact of alcohol consumption on QoL in older people are scarce and disparate. Chan et al. [18], in a study of 1594 people aged 50–97, found that regular alcohol consumption is associated with better QoL. However, other studies have found no significant relationship between alcohol consumption and QoL in this age group [19,20]. In a study conducted in an older Spanish population of 2163 people over 60 years of age, a positive relationship was initially observed between alcohol consumption (both moderate and severe) and health-related QoL, more specifically in the physical health component. However, in the follow-up conducted three years and three months later, this relationship was not significant [21]. Kaplan et al. [22] conducted a prospective study in Canada with 5404 people over 50, analyzing different consumption patterns over 14 years. Their results indicate that drinkers with a moderate persistent pattern were those with higher initial levels of health-related QoL. However, the decline in health-related QoL is similar for all drinking patterns except for those who decreased their consumption, where QoL is better.

The MentDis_ICF65+ Study (health and well-being of people aged 65–84 in Europe) [23,24,25,26,27,28,29,30,31] is a cross-cultural and longitudinal study conducted in six European cities on the prevalence of mental disorders in older people, as well as other aspects such as the relationship between the physical and mental health of older people and their QoL. This study aims to fill the research gap about the relationship of any kind of mental disorder (not only emotional disorders) with QoL, using a standardized diagnostic interview of mental health adapted to people over 65 years old. This study has been funded by a grant from the European Commission (Grant No.: 223105) within the 7th EU Research Framework Program, and in Spain the study has been conducted from the Faculty of Psychology of the Complutense University of Madrid. The main results of the MentDis_ICF65+ Study are presented regarding the relationship between QoL and the following variables: gender, age and physical and mental health of people over 65 years old in the Community of Madrid.

## 2. Materials and Methods

### 2.1. Design

All interviews were conducted in people’s homes. Participants gave their written consent before the interview. The average length of the interviews was 84 min. The full procedure can be found in Andreas et al. [23]

The Ethics Committee of the Complutense University of Madrid (No. 22032010) approved both the sample collection procedure and the way in which participants gave their informed consent to participate in the study.

### 2.2. Participants

The sample (*N* = 555) was taken in the Community of Madrid, both in the 21 districts of Madrid city and in rural areas. The sample, weighted by age and sex, is made up of men and women aged between 65 and 84. The criteria for being included in the sample were the following: (a) ability to give informed consent to participate in the study; (b) living in the Community of Madrid; and (c) being between 65 and 84 years old. The criteria for exclusion from the sample were as follows: (a) presence of moderate cognitive impairment assessed through a Spanish adaptation [32] of the Mini Mental State Examination (Mini-mental [33]) using as a cut-off point of >18; and (b) insufficient language communication skills to be interviewed. The sample was randomly selected from postal addresses of residents in Madrid from a private market research company, and the Madrid City Council. Participants were approached with a written invitation letter and a phone call. The total number of contacted people was 3375. The total number of people who agreed to participate was 584 (17.3%). Three participants were excluded for having cognitive problems. A total of 26 people dropped out for any of the following reasons: withdrawal of willingness to participate; not reached; illness; and invalid interview (incomplete data). To assess the comparability of the recruited sample with the general community population from Madrid, the following descriptive comparisons were made: MentDis65+ sample vs. catchment area and vs. country population. The comparison data were obtained from the Office for National Statistics, Census 2011; Madrid (Spain)—Instituto Nacional de Estadistica, Population and Housing Census 2011 (www.ine.es). The following sociodemographic variables were compared: work status, marital status, number of children, education, number of household members and place of birth.

Table 1 shows the main socio-demographic characteristics of the sample. The average age of the sample was 73.5 years. In terms of marital status, more than half of the sample was married at the time of the interview, and a quarter was widowed.

### 2.3. Variables and Instruments

The following variables and instruments were included in the assessment:

- Socio-demographic variables: The interview includes a form for collecting socio-demographic data: sex, age, marital status, employment situation, years of schooling, number of significant close relationships, living alone, degree of burden borne, financial situation, frequency of financial problems, religious affiliation and number of illnesses or physical symptoms.
- Mental disorders: The MentDis_ICF65+ team developed the International Composite Diagnostic Interview for people over 65 (CIDI65+, [31]). The CIDI65+ is adapted to the skills and needs of older people in terms of social, cognitive and psychological aspects. CIDI65+ provides diagnoses according to the criteria of the DSM-IV-R classification system [34]. The assessment of the test–retest reliability of the newly adapted CIDI65+ showed good results ranging between k = 0.55 for major depression and k = 1.00 for obsessive-compulsive disorder (k = 1.00). Intraclass correlation coefficients for the age of onset, recency, quantity, frequency and duration questions ranged between k = 0.60 and 0.90. Further details of the CIDI65+ psychometric properties are reported in Wittchen et al. [31].

In this study, a difference is established between mental disorders and physical disorders. In this regard, it should be clarified that a physical disorder is a medical disorder that has a mechanical test available (such as chemical tests or brain scans), whereas a mental disorder has no laboratory or imaging tests and is diagnosed by a behavioral syndrome (like DSM).

- Quality of life: A reduced version of the World Health Organization Quality of Life BREF scale was used to assess quality of life (WHOQoL-BREF, [35]). It consists of eight items, with five Likert-type answer options, asking the subject about different aspects of quality of life during the past month, and includes two additional global questions: “How would you rate your quality of life” and “How satisfied are you with your health?” These ten items of the reduced version of the WHOQoL BREF scale assess satisfaction in some aspects of life. The items are grouped into 3 subscales: Physical health, Social relations and Environment.

### 2.4. Analysis

Mean score analyses of the different instruments used were carried out to find out the average scores of the people in the sample regarding QoL. A variance analysis (ANOVA) was carried out to find out the possible statistically significant differences between the different groups of subjects in QoL measures (WHOQoL-BREF). A multiple linear regression analysis using the “stepwise” method was performed to determine the effect of age, gender, other socio-demographic factors and the presence of mental disorders on QoL (WHOQoL-BREF). Subsequently, a multiple linear regression analysis using the stepwise method was carried out to determine the effect of age (by 65–74- and 75–84-year-old strata), gender and the presence of mental disorders on QoL (WHOQoL-BREF). All analyses were conducted using SPSS version 22.0 (IBM Corp., New York, NY, USA) [36].

## 3. Results

### 3.1. Quality of Life of People Over 65 and Its Relationship to Gender and Age Variables

Most people in the sample report a fairly good level of QoL, with an average score of 65.12 out of 100 (with a standard deviation of 17.57) on the overall WHOQoL-BREF scale. Table 2 contains details of the average score of the ten items on the WHOQoL BREF scale that assess satisfaction in some aspects of life, showing the differences according to gender and dividing the sample into two age groups (65–74 and 75–84 years of age). It is observed that women score higher than men on the two items related to personal relationships: item 5—“How satisfied are you with your personal relationships?”, and item 6—“How satisfied are you with the support you get from your friends?” (4.19 and 4.05 in women vs. 4.06 and 3.87 in men), and consequently score significantly higher than men on the Social relations subscale (77.94 in women vs. 74.11 in men). On the other hand, men obtain a significantly higher average score in item 4—“How satisfied are you with your working ability?” (3.88 in women vs. 4.05 in men), and in item 10—“How satisfied are you with your health?” (3.46 in women vs. 3.72 in men), as well as a higher, and therefore better, overall quality of life score than women (62.63 in women vs. 67.04 in men).

Regarding differences according to age, the oldest group (75–84 years of age) obtains significantly lower average scores than the youngest group in the three subscales: Physical health (70.65 in the 65–74 group compared to 65. 45 in the 75–84 group), Environment (72.15 in the 65–74 group against 69.88 in the 75–84 group) and Social relations (77.84 in the 65–74 group against 74.07 in the 75–84 group), as well as in item 4—“How satisfied are you with your working ability?” (4.06 in the 65–74 group against 3.85 in the 75–84 group), 6—“How satisfied are you with the support you get from your friends?” (4.09 in the 65–74 group vs. 3.82 in the 75–84 group), and 8—“How satisfied are you with your transport? (4.13 in the 65–74 group vs. 3.95 in the 75–84 group), plus a significantly higher score in item 1—“How well do you need medical treatment to function in your daily life?” (2.59 in the 65–74 group vs. 2.88 in the 75–84 group). There are no significant differences between the two age groups in the total score.

### 3.2. Comparison of the Impact of Physical and Mental Disorders and Symptoms On QoL

The differences in the overall results of the WHOQoL-BREF scale were examined by dividing the sample into four groups:

Group 1 (Mental Disorder): People with mental disorders (excluding nicotine addiction) and who did not report having significant physical problems.

Group 2 (Physical Disorder): people who had two or more physical health-related problems, such as persistent pain, recurrent pain, weakness, heart problems, hypertension, diabetes, gastrointestinal problems, respiratory problems, cancer, incontinence, physical disability, hearing problems, poor vision or neurological diseases. Only people with at least two physical symptoms were included as the vast majority of the sample had at least one of them.

Group 3 (Both): people who score positive for both problem types (physical and mental) are included.

Group 4 (None): subjects do not score positive for either physical or mental problems.

The variance analysis (ANOVA) shows statistically significant differences between the different groups on the WHOQoL BREF scale = 25.11, *p* < 0.001. The mean scores on the WHOQoL BREF scale by type of disorder are included in Table 3. On the WHOQoL BREF scale, people who had both physical health problems and mental disorders (Group 3, Both) obtained a significantly lower mean score than those who had only physical problems (Group 2, Physical Disorder) or only a mental disorder (Group 1, Mental Disorder), or neither (Group 4, None).

Table 4 shows the comparison of the impact of physical and mental disorders on the WHOQoL BREF scale, including the means for each group. The presence of physical problems implies a significantly lower average score in QoL than that of people who have neither physical nor mental problems (Group 4, None), as well as those who have a mental disorder (Group 1, Mental Disorder).

### 3.3. Quality of Life in People over 65 in the Community of Madrid and Its Relationship with Socio-Demographic Variables and Physical and Mental Health

The socio-demographic variables included in the analysis were the following: sex, age, marital status, employment situation, years of schooling, number of significant close relationships, living alone, degree of burden borne, financial situation, frequency of financial problems, religious affiliation and number of physical illnesses or symptoms. To see the effect of mental health, we included the existence of any depressive, anxiety or alcohol abuse/dependence disorder in the last year (CIDI65+), the presence of any psychological disorder (excluding nicotine) in the last year (CIDI65+) and the number of psychological symptoms from CIDI65+ List A5 (sadness, depression, demoralization, anxiety, worry, nervousness, sleep problems, nightmares, problems with alcohol, problems with medication, fatigue, sexual problems, appetite or weight problems, thoughts of suicide, thoughts of terrible events, confusion, low self-esteem, irritability, insecurity, difficulties in making decisions, anger and aggression, strange thoughts and experiences or sensations).

The final model obtained for the WHOQoL BREF scale is shown in Table 5. The regression model for the dependent variable “WHOQoL BREF Scale” explains 41.43% of the variance (*R*^2^ = 0.413). In the total WHOQoL BREF Scale score, a higher number of physical and psychological symptoms, experiencing financial difficulties and the presence of some psychological disorder are associated with a worse quality of life, while continuing to work has a positive effect on it. Regarding the three subscales (Physical health, Social relations and Environment), there are some differences in the variables that have the greatest impact on the results. The presence of a greater number of problems related to physical health has a negative impact on both the Physical health and Environmental scales, while a greater number of psychological symptoms significantly affect the three subscales. The presence of a depressive disorder leads to worse scores on the Physical health scale, as well as to a poor financial situation. In the Social relations subscale, being widowed, separated or divorced (as compared to being married or never married) has a negative effect, while in the Environmental subscale, considering religion as little or not important implies worse scores. Regarding the variables that have a positive impact on the different subscales, being a woman is related to better scores in the Social relations subscale, and continuing to work has a positive impact on both the Environmental and Social relations subscales.

## 4. Discussion

Most of the over-65s in the sample report a fairly acceptable level of QoL, with an average score of 65.12 out of 100 (with a standard deviation of 17.57) on the overall WHOQoL-BREF scale. These results are in line with those found in previous studies [9,37]. As regards gender differences, the scores obtained by women on the WHOQoL-BREF scale indicate that they have worse QoL than men in the sample. These data are in line with a previous study carried out with people over 65 in the Spanish population [7] and could be explained by the greater burden of care for dependents assumed by women over 65 compared to men of the same generation. Only in the area of social relations on the WHOQoL-BREF scale do the women in the sample have good or even better scores than men. These data could be explained by the high participation of women in the socio-cultural offer in the Community of Madrid. This study highlights that QoL decreases with age, which coincides with the results found in the European Health Survey in Spain 2014 of the National Institute of Statistics [38].

Regarding other socio-demographic variables, the results obtained indicate that a more precarious economic situation is related to a worse QoL, as some studies had previously shown [39]. The employment situation also seems to have an impact, with better QoL scores observed for those people who continue to work, although this could be due to the fact that they belong to the youngest age group in the sample. On the other hand, working as a housewife, in comparison with people who are retired or who continue to be active in the labor market, also has a negative impact on QoL.

Results comparing the consequences of physical and mental disorders in QoL indicate that both disorders have a similar impact on the QoL of people over 65. Regarding the differences in QoL between people with and without a mental disorder, the results indicate that people with a mental disorder show a worse QoL than people without. Moreover, the results also indicate, on the one hand, that the WHOQoL BREF scale provides accurate data on the impact of physical problems, which is moderate, and, on the other hand, the sum of physical and mental problems of Group 3 (Both), with this group presenting a lower QoL than people from the rest of the groups.

These results show that not only does the existence of a mental disorder affect QoL, but also the presence of a greater number of psychological symptoms (without necessarily meeting the criteria for a mental disorder) is a predictor of worse QoL. There are several studies on this subject that find how the presence of depressive or anxious symptoms (without necessarily meeting the diagnostic criteria of a mental disorder) has a negative impact on the QoL of older people [40,41,42,43,44]. These data are especially relevant since the psychological symptoms of common mental disorders (anxiety disorders, mood disorders, somatization and adaptive disorders) are the most frequently found in primary care [45] and 39% of people with a common disorder who attend primary care do not receive any treatment (PsicAP—Psychology in Primary Care clinical study [46]) when it has been shown that such psychological symptoms negatively affect their QoL. Regardless of whether the diagnostic criteria for a given disorder are met, it is necessary to address the psychological symptoms and emotional problems in the lives of people over 65.

These results show that mental health has as much of a burden on the QoL of people over 65 as physical health does. If an older person suffers from both health problems (mental and physical), their QoL worsens exponentially.

The advantages and limitations of this study should be noted. An advantage of the present study is the use of a standardized and structured instrument to assess mental disorders, adapted to the characteristics of older people [31]. The representativeness of the sample indicates that there are limitations related to the definition of the criteria for the exclusion of the sample used in this study. People with a severe cognitive impairment, those living in a residence, homeless people and non-Spanish speaking people have not been included. Future studies should include people who live in a residence or those with dementia. Moreover, the results are based on DSM-IV-TR criteria and using DSM-5 diagnoses could have led to different results. Given the fact that the core features have remained the same for the evaluated disorders, very different prevalence estimations would not be expected.

## 5. Conclusions

This study has highlighted the need to address the emotional aspects of people over 65, as they have a direct impact on their QoL, especially in the case of older women. Since people with mental health problems themselves are often reluctant to seek help from health professionals [47], the latter should strive to employ strategies to detect mental health problems in people over 65 [48].

This detection of psychological symptoms, taking into account the gender perspective as few studies have examined the mental health of minority genders in later life, will make it possible to deal with these emotional problems before they affect the QoL of the people who suffer from them.

In conclusion, in our opinion, to improve the access to health services of older people with a mental disorder, it is important to (1) improve the detection of mental disorders in older people by primary care professionals. The PsicAP study proposes the use of different versions of the Patient Health Questionnaire, such as the PHQ-9, PHQ-4, PHQ-PD and the GAD-7; (2) reduce the stigma of professionals towards older people with mental disorders; (3) combat self-referenced stigma and ageism; and (4) provide adequate social and health services for older people with a mental health problem.

## Figures and Tables

**Table 1:** Socio-demographic characteristics of the sample.

	Total (*N* = 555)
Age, *n* (%)	
- 65–69	184 (33.2%)
- 70–74	112 (20.2%)
- 75–79	147 (26.5%)
- 80–84	112 (20.2%)
Sex, *n* (%)	
- Women	288 (51.9%)
- Men	267 (48.1%)
Born in Spain	532 (95.9%)
Marital status	
- Married	336 (60.5%)
- Separated/Divorced/Widowed	192 (34.6%)
- Never married/others	27 (4.9%)
Years of schooling	
- From 0 to 8	302 (54.4%)
- From 9 to 12	124 (22.3%)
- 13 or more	127 (22.9%)
School graduate	
- Yes	297 (53.5%)
- No	258 (46.5%)
Religious affiliation	
- Very important	186 (33.5%)
- Something important	168 (30.3%)
- Not very important	127 (22.9%)
- Nothing important	74 (13.3%)
Employment situation	
- Retirees	400 (72.1%)
- Housewife	137 (24.7%)
- Working	13 (2.3%)
- Unemployed	4 (0.7%)
- Others	1 (0.2%)
Financial situation	
- Very good	15 (2.7%)
- Good	150 (27%)
- Enough	291 (52.4%)
- Poor	86 (15.5%)
- Very poor	13 (2.3%)

**Table 2:** Average scores for each of the ten items on the WHOQoL BREF scale that assess satisfaction in some aspects of life.

Item	Total (1–5)	Women	Men	65–74	75–84
1. How much do you need any medical treatment to function in your daily life?	2.72 ± 1.22	2.79 ± 1.29	2.65 ± 1.14	2.59 ± 1.19 *	2.88 ± 1.24 *
2. How safe do you feel in your daily life?	3.45 ± 0.78	3.40 ± 0.80	3.49 ± 0.76	3.48 ± 0.82	3.41 ± 0.73
3. How satisfied are you with your ability to perform your daily living activities?	3.95 ± 0.82	3.92 ± 0.86	3.98 ± 0.78	4.00 ± 0.81	3.89 ± 0.83
4. How satisfied are you with your capacity for work?	3.96 ± 0.78	3.88 ± 0.83 *	4.05 ± 0.73 *	4.06 ± 0.78 *	3.85 ± 0.77 *
5. How satisfied are you with your personal relationships?	4.13 ± 0.70	4.19 ± 0.70 *	4.06 ± 0.70 *	4.14 ± 0.73	4.11 ± 0.67
6. How satisfied are you with the support you get from your friends?	3.96 ± 0.82	4.05 ± 0.85 *	3.87 ± 0.78 *	4.09 ± 0.78 *	3.82 ± 0.84 *
7. How satisfied are you with your access to health services?	4.04 ± 0.84	4.03 ± 0.90	4.05 ± 0.77	4.05 ± 0.86	4.03 ± 0.82
8. How satisfied are you with your transport?	4.05 ± 0.78	4.01 ± 0.84	4.08 ± 0.70	4.13 ± 0.80 *	3.95 ± 0.74 *
9. How would you rate your quality of life?	3.60 ± 0.76	3.55 ± 0.78	3.64 ± 0.74	3.57 ± 0.78	3.63 ± 0.73
10. How satisfied are you with your health?	3.59 ± 0.95	3.46 ± 0.99 *	3.72 ± 0.88 *	3.62 ± 0.93	3.54 ± 0.97
Physical health subscale (0–100)	68.23 ± 18.46	66.84 ± 19.50	69.71 ±17.20	70.65 ± 18.00 *	65.45 ± 18.63 *
Environment subscale (0–100)	71.09 ± 13.25	70.38 ± 14.01	71.85 ± 12.38	72.15 ± 13.85 *	69.88 ± 12.44 *
Social relationships subscale (0–100)	76.09 ± 16.34	77.94 ± 16.44 *	74.11 ± 16.03 *	77.84 ± 16.39 *	74.07 ± 16.08 *
Global score (0–100)	64.76 ± 18.26	62.63 ± 18.72 *	67.04 ± 17.51 *	65.54 ± 17.88	64.64 ± 17.44

* *p* < 0.05, comparison between age groups 65–74 and 75–84.

**Table 3:** Mean scores by type of disorder on the WHOQoL Bref * global scale.

Type of Disorder	WHOQoL Bref Global
Group 1.	68.75 [65.18; 72.32]
Mental Disorder	*n* = 42
Group 2.	62.94 [60.98; 64.90]
Physical Disorder	*n* = 254
Group 3.	57.44 [54.06; 60.81]
Both	*n* = 116
Group 4.	74.54 [71.55; 77.54]
None	*n* = 137

* ANOVA = 25.11 (*p* < 0.001).

**Table 4:** Comparison of the impact of physical and mental disorders on the WHOQoL BREF scale: averages for each group: analysis of variance; mean differences; statistical significance.

	G1 Mental Disorder	G2 Physical Disorder	G3 Both	G4 None
		dif. M (sig.)	dif. M (sig.)	dif. M (sig.)
G1. Mental Disorder		5.80 (0.028) *	11.31 (0.001) **	−5.79 (0.067)
G2. Physical Disorder	−5.81(0.028) *		5.51 (0.029) *	−11.60 (0.000) **
G3. Both	−11.31 (0.000) **	−5.51 (0.029) *		−17.11 (0.000) **
G4. None	5.79 (0.067)	11.60 (0.000) **	17.11 (0.000) **	

ANOVA * *p* < 0.05; ** *p* < 0.001.

**Table 5:** Regression model for the dependent variable “WHOQoL BREF Scale”.

Variable	B	Standard Error	β	Sig.
Number of psychological symptoms	−2.87	0.43	−0.31	0.000
Number of physical symptoms	−1.51	0.28	−0.26	0.000
Financial situation	−5.35	0.94	−0.24	0.000
Any psychological disorder	−3.70	1.55	−0.10	0.017
Employment situation: working	10.34	4.76	0.09	0.031

*p* < 0.05.
